# Mathematical modeling for Phase I cancer trials: A study of metronomic vinorelbine for advanced non-small cell lung cancer (NSCLC) and mesothelioma patients

**DOI:** 10.18632/oncotarget.17562

**Published:** 2017-05-02

**Authors:** Fabrice Barlesi, Diane-Charlotte Imbs, Pascale Tomasini, Laurent Greillier, Melissa Galloux, Albane Testot-Ferry, Mélanie Garcia, Xavier Elharrar, Annick Pelletier, Nicolas André, Céline Mascaux, Bruno Lacarelle, Raouf El Cheikh, Raphaël Serre, Joseph Ciccolini, Dominique Barbolosi

**Affiliations:** ^1^ Aix Marseille University, APHM, Marseille Early Phases Cancer Trials Center CLIP, Marseille, France; ^2^ Aix Marseille University, SMARTc Unit, INSERM U911, Marseille, France; ^3^ Aix Marseille University, APHM, Department of Pharmacology, Marseille, France; ^4^ Aix Marseille University, APHM, Department of Pediatric Hematology and Oncology, Marseille, France

**Keywords:** lung cancer, mesothelioma, mathematical modeling, vinorelbine, metronomic

## Abstract

**Introduction:**

Using mathematical modelling allows to select a treatment's regimen across infinite possibilities. Here, we report the phase I assessment of a new schedule for metronomic vinorelbine in treating refractory advanced NSCLC and mesothelioma patients.

**Results:**

Overall, 13 patients were screened and 12 were treated (50% male, median age: 68yrs), including 9 NSCLC patients. All patients received at least one week (3 doses) of treatment. At data cut-off, the median length of treatment was 6.5 weeks (1–32+). All the patients presented with at least one adverse event (AE) and six patients with a severe AE (SAE). One partial response and 5 stable diseases were observed. The median OS was 6.4 months (95% CI, 4.8 to 12 months). The median and mean vinorelbine's AUC were 122 ng/ml*h and 159 ng/ml*h, respectively, with the higher plasmatic vinorelbine exposure associated with the best ORR (difference of AUC comparison between responders and non-responders, *p-value* 0.017).

**Materials and Methods:**

The mathematical modelling determined the administration of vinorelbine, 60 mg on Day 1, 30 mg on Day 2 and 60 mg on Day 4 weekly until progression, as the best schedule. Advanced NSCLC or mesothelioma patients progressing after standard treatment were eligible for the trial. NCT02555007.

**Conclusions:**

Responses with acceptable safety profile were observed in heavily pretreated NSCLC and mesothelioma patients using oral vinorelbine at this metronomic dosage based on a mathematic modeling. This study demonstrates the feasibility of this new type of approach, as mathematical modeling may help to rationally decide the better regimen to be clinically tested across infinite possibilities.

## INTRODUCTION

Vinorelbine is a semi-synthetic vinka-alkaloid with activity in several cancer, including NSCLC and mesothelioma. Vinorelbine is available both as intravenous and oral forms. The oral form allows the regular administration of vinorelbine at less-toxic doses over prolonged periods of time (metronomic chemotherapy) [1]. Using metronomic chemotherapy should theoretically allow to reduce the incidence of treatment-related toxicities, while possibly offering additional mechanisms of actions such as anti-angiogenic or immuno-stimulating effects [2]. However, determining the optimal scheduling and dosing of metronomic regimen remains a challenge as numerous combinations of small doses, time of administration and treatment's durations are possible. As a consequence, several regimens have been tested, mostly designed on a trial-and-error mode. Of note, the vinorelbine regimens substantially vary across previous studies with, doses ranging from 30 to up to 70 mg, administration ranging from continuous to weekly schedules, and a wide range for durations of treatment [3]. Finally, the dose and schedule for metronomic vinorelbine have been assessed in four phase I trials and a 50 mg dosage on Days 1, 3 and 5 each week was the recommended phase 2 dose [4–7]. However, this final choice was based, firstly on an empirical choice for days 1, 3 and 5 administrations (mainly based on the theoretical 48 hours half-life of the drug) [8]. and secondly, on a classical maximal tolerated dose (MTD) approach, which is not ideal when minimizing toxicities is expected. In this respect, developing model-driven approaches for metronomic chemotherapy is an attractive strategy [9]. We therefore used mathematical modelling to determine a new schedule for the metronomic administration of vinorelbine. The mathematical model was set up to improve efficacy while reducing toxicity and proposed the vinorelbine 60 mg on Day 1, 30 mg on Day 2 and 60 mg on Day 4 as the best schedule [10]. We report here the phase I assessment of this new schedule of metronomic vinorelbine.

## RESULTS

Overall, from Aug 26, 2015 to Feb 18 2016, 13 patients have been screened and 12 patients treated (one screen failure). The patients’ characteristics are summarized in Table 1.

**Table 1:** Patients’ characteristics

Characteristics	*n*
**Gender (Male/Female)**	6/6
**Age (Median, Range), yrs**	68 (44–78)
**Tumor type**	
** Advanced NSCLC (Stage IIIB/Stage IV)**	0/9
** Mesothelioma**	3
**Previous lines of therapy (Median, Range)**	4 (1–7)
**Best objective response to the latest line of therapy**	
** Progression**	9
** Stable disease**	2
** Partial response**	1

All the patients received at least one week of oral vinorelbine 60 mg Day 1, 30 mg Day 2 and 60 mg Day 4. At the data cut-off (Sep 1st 2016), one patient was still on treatment and the median length of treatment was 6.5 weeks (range, 1 to 32 weeks).

All the patients presented with at least one adverse event (AE). The description of the AEs is reported in Table 2. Six patients presented with a severe AE (SAE). One patient died as a consequence of a drug-unrelated SAE (pulmonary infection).

**Table 2:** Adverse events

	Summary of AEs					
	All grades	Grade 1	Grade 2	Grade 3	Grade 4	Grade 5
**All AE**	12	2	3	3	3	1
**Related to treatment**	12	3	2	4	3	0
**Description of AEs**						
	**Hematological and biological AEs**					
	**All grades**	**Grade 1**	**Grade 2**	**Grade 3**	**Grade 4**	**Grade 5**
**Anemia**	10	3	6	1	0	0
**Leucopenia**	8	1	4	2	1	0
**Neutropenia**	5	0	1	1	3	0
**Lymphopenia**	9	3	4	1	1	0
**Thrombopenia**	2	2	0	0	0	0
**Hyperleukocytosis**	4	4	0	0	0	0
**Thrombocytosis**	6	6	0	0	0	0
**Hypoalbuminemia**	3	1	2	0	0	0
**GGT increased**	2	2	0	0	0	0
**Hyponatremia**	2	2	0	0	0	0
**Hyperglycemia**	2	2	0	0	0	0
**Vitamin D discreased**	1	1	0	0	0	0
**Creatinine increased**	1	1	0	0	0	0
**Hyperalbuminemia**	1	1	0	0	0	0
**Hypercalcemia**	1	1	0	0	0	0
**Hyperkaliema**	1	1	0	0	0	0
**Hypochloronatremia**	1	1	0	0	0	0
**Hypoprotidemia**	1	1	0	0	0	0
**Clinical AEs**						
	**All grades**	**Grade 1**	**Grade 2**	**Grade 3**	**Grade 4**	**Grade 5**
**Asthenia**	7	4	3	0	0	0
**Anorexia**	2	1	1	0	0	0
**Fever**	2	2	0	0	0	0
**Febrile neutropenia**	1	0	0	1	0	0
**Infection and sepsis**	2	1	0	0	0	1
**Sweating**	1	1	0	0	0	0
**Constipation**	8	8	0	0	0	0
**Nausea**	7	5	2	0	0	0
**Vomiting**	6	5	0	1	0	0
**Abdominal pain**	5	4	1	0	0	0
**Diarrhea**	3	3	0	0	0	0
**Headache**	2	2	0	0	0	0
**Cough**	3	2	1	0	0	0
**Arythmia**	2	2	0	0	0	0
**Pulmonary embolism**	1	0	0	1	0	0
**Lower limbs oedema**	2	2	0	0	0	0
**Mucitis**	2	2	0	0	0	0
**Skin Rash**	1	1	0	0	0	0
**Chills**	1	1	0	0	0	0
**Myalgia**	1	1	0	0	0	0
**Anxiety**	1	1	0	0	0	0

**Figure 1:**
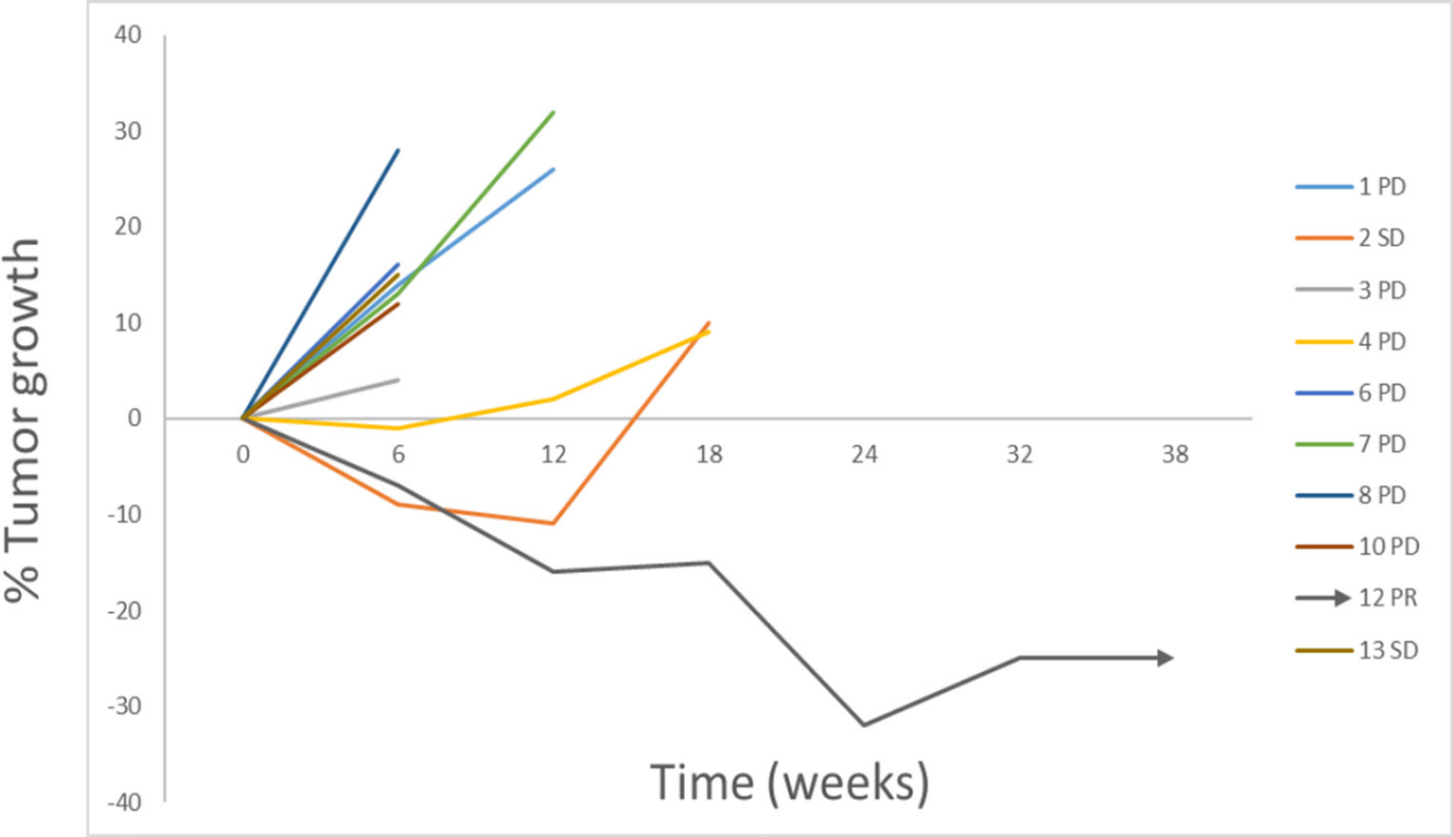
Illustration of the changes in tumor burden over time since treatment initiation for each of the evaluable patients

Two patients were not evaluable for response (dropped out of the study before the first radiological assessment). The ORR are reported in Table 3 and Figure 1. After a median follow-up of 12.1 months for alive patients, the median PFS and OS were 2.5 months (95% CI, 1.2 to 7.6 months) and 6.4 months (95% CI, 4.8 to 12 months), respectively, with three patients still alive.

**Table 3:** Summary of the best radiological response globally and by tumor type

Best response	NSCLC	Mesothelioma	Global
**Partial Response**	1	0	1
**Stable Disease**	3	2	5
**Progression**	3	1	4

A three compartments model best described oral vinorelbine PK data [10]. Considering the continuous administration of the metronomic dosing regimen, the relationship between the vinorelbine concentration and the neutrophils rate was adequately described by an adapted Friberg's model [11]. The median and mean vinorelbine's AUC were 122 ng/ml*h and 159 ng/ml*h, (range 13–392 ng/ml*h and 95% confidence interval (CI 95%) of 86–233 ng/ml*h), respectively. However, large variations were seen across the patients’ population. In addition, PK/PD analysis shows that the patients with the higher plasmatic vinorelbine exposure where those presenting with the best ORR (AUC comparison between responders and non-responders shows a statistically significant difference *p-value* = *0.017*).

The mathematical model considering PK, AEs and ORR from these 12 patients was upgraded in order to define an optimized vinorelbine protocol aiming to reach higher efficacy with at least similar safety profile. The *In silico* simulations confirmed that no better schedule, respecting both minimization of (hematological) side effects and maximization of response, could be calculated. Therefore, 20 additional patients will be treated with the same metronomic vinorelbine protocol.

## DISCUSSION

The support of mathematical modelling is a potential tool to optimize cancer treatments. Indeed, the number of available therapeutic options (i.e., drugs, sequential or concomitant combinations, time and dosage for the administration) exceeds available resources for empirical testing. In the field of metronomics, outdated and underpowered strategies have generated conflicting results regarding the best way to administrate many drugs such as gemcitabine, vinorelbine, or cyclophosphamide [9]. In particular, the issue of PK variability has been underestimated for metronomic regimens [12, 13]. So far to our knowledge, this study provides the first example of the assessment of a computational metronomic schedule, presently for oral vinorelbine. This study demonstrates the feasibility of this approach since both preliminary encouraging signs of activity and no unexpected toxicities were observed, in this heavily pre-treated patients’ population. Of note, this study also highlight the large inter-individual variability observed in the PK of oral vinorelbine. Several reasons can explain this level of discrepancy in the plasma exposure to the drug including nutritional status (food effect, cachexia), comorbidities impacting on renal and liver functions, and lack of control for possible drug-drug interactions in outpatients. Despite this inter-patient variability, prolonged responses with an acceptable safety profile seem possible.

Metronomic chemotherapy has suggested efficacy in several settings, including breast and NSCLC patients [14]. The limited number of patients included in the study part one does not provide with another option to replace the currently used schedule. However, the mathematical model will be further improved by including its effect on the immune system on the basis of the work of Serre *et al*. [15] and enrichment with data collected in the part two of the study as well as in other clinical trials based on metronomic vinorelbine [16].

In summary, responses with acceptable safety were observed in heavily pretreated patients with NSCLC and mesothelioma using oral vinorelbine at metronomic dosage based on a mathematic modeling. This study demonstrates the feasibility of this new type of approach, as mathematical modeling may help to rationally decide across infinite designs the better to be clinically tested.

## MATERIALS AND METHODS

The whole protocol has been previously published [10]. Briefly, patients with proven advanced NSCLC or mesothelioma progressing after standard treatments were eligible. The main eligibility criteria were age over 18 years, ECOG PS 0–2, adequate hematological, liver and renal functions, and presence of at least one measurable lesion. The main exclusion criteria were uncontrolled cardiac disease, active infection, and previous history of cancer.

The study was approved by the ethics committee (CPP Marseille 1, on 14 April 2015) and the National Agency for the Security of Drugs (ANSM, on 26 May 2015). All patients signed an informed consent form. The study was registered with EudraCT 2015-000138-31 and ClinicalTrials.gov ID: NCT02555007.

### The pre-protocol mathematical modelling was divided into 3 parts

The pharmacokinetic (PK) model was sought to describe the evolution over time of vinorelbine blood concentrations administrated orally. This model was driven by pharmacokinetic parameters reflecting the inter-individual variability. These parameters can be estimated for each individual using Bayesian method and population-based approaches.

The pharmacodynamic (PD) safety model describing the impact of drug concentrations on the hematopoietic chain, mainly resulting in neutropenia for vinorelbine. This model required modifications of the initial Friberg model [11] considering continuous administrations of metronomic dosing regimen.

The PD efficacy model describing the action of drug on both tumor and endothelial cells, as well as the emergence of resistant clones throughout time. This model was built from the following hypotheses: H1, In absence of treatment, tumor growth follows a Gompertz model; [17] H2, Endothelial cells are more sensitive to chemotherapy agents than cancer cells; [18] H3, Depleting endothelial cells will affect tumor growth; [19] and H4, Endothelial cells are more genetically stable than cancer cells and therefore less likely to develop resistance to chemotherapy agents.

Therefore, patients were treated continuously with oral vinorelbine at 60 mg on Day 1, 30 mg on Day 2 and 60 mg on Day 4, with adequate antiemetic medication. The treatment was continued until disease progression or unacceptable toxicity. A bi-weekly meeting was conducted to analyze the adverse events and decide the study's continuation.

Patients were assessed for response (ORR) at baseline and every 6 weeks, by RECIST 1.1 or modified RECIST (mesothelioma patients). Individual pharmacokinetic (PK) analysis was based on one set of four blood samples four days after treatment initiation and assessed from plasma measurement of vinorelbine after LC-MS/MS analysis. The limit of quantification was 0.1 ng/mL and the Coefficient of variation (CV) for precision using spiked plasma control samples was lower than 15% for each quality control. PK/pharmacodynamic (PK/PD) analyses were done using Monolix 4.3.3 (Lixoft SAS).

The study was designed as a two-stage approach. For the first stage, the sample size allowing to consolidate the calibration of the mathematical model parameters was calculated to be based on 12 patients. Data generated in the first part of the trial were then computed to either validate the relevance of the pre-defined schedule of administration or propose a different optimized protocol that would then have to be assessed in the second stage of the study with a validation sample of 20 patients.
